# Prostate volume analysis in image registration for prostate cancer care: a verification study

**DOI:** 10.1007/s13246-023-01342-4

**Published:** 2023-10-11

**Authors:** Jessica M. Bugeja, Georges Mehawed, Matthew J. Roberts, Nicholas Rukin, Jason Dowling, Rebecca Murray

**Affiliations:** 1 Australian e-Health Research Centre, Commonwealth Scientific and Industrial Research Organisation, Health and Biosecurity, Herston, Australia; 2 Herston Biofabrication Institute, Urology Program, Herston, Australia; 3 Urology Department, Redcliffe Hospital, Redcliffe, Australia; 4 School of Medicine, The University of Queensland, Brisbane, Australia; 5 Australian Institute of Bioengineering and Nanotechnology, The University of Queensland, Brisbane, Australia; 6 Urology Department, Royal Brisbane and Women’s Hospital, Herston, Australia; 7 University of Queensland, University of Queensland Centre for Clinical Research, Herston, Australia; 8 School of Information Technology and Electrical Engineering, The University of Queensland, Brisbane, Australia

**Keywords:** Prostate, Magnetic resonance imaging, Computed tomography, Image registration, Cancer care, Urology

## Abstract

Combined magnetic resonance imaging (MRI) and positron emission tomography/computed tomography (PET/CT) may enhance diagnosis, aid surgical planning and intra-operative orientation for prostate biopsy and radical prostatectomy. Although PET-MRI may provide these benefits, PET-MRI machines are not widely available. Image fusion of Prostate specific membrane antigen PET/CT and MRI acquired separately may be a suitable clinical alternative. This study compares CT-MR registration algorithms for urological prostate cancer care. Paired whole-pelvis MR and CT scan data were used (n = 20). A manual prostate CTV contour was performed independently on each patients MR and CT image. A semi-automated rigid-, automated rigid- and automated non-rigid registration technique was applied to align the MR and CT data. Dice Similarity Index (DSI), 95% Hausdorff distance (95%HD) and average surface distance (ASD) measures were used to assess the closeness of the manual and registered contours. The automated non-rigid approach had a significantly improved performance compared to the automated rigid- and semi-automated rigid-registration, having better average scores and decreased spread for the DSI, 95%HD and ASD (all *p* < 0.001). Additionally, the automated rigid approach had similar significantly improved performance compared to the semi-automated rigid registration across all accuracy metrics observed (all *p* < 0.001). Overall, all registration techniques studied here demonstrated sufficient accuracy for exploring their clinical use. While the fully automated non-rigid registration algorithm in the present study provided the most accurate registration, the semi-automated rigid registration is a quick, feasible, and accessible method to perform image registration for prostate cancer care by urologists and radiation oncologists now.

**Supplementary Information:**

The online version contains supplementary material available at 10.1007/s13246-023-01342-4.

## Introduction

Prostate specific membrane antigen (PSMA) positron emission tomography/computed tomography (PET/CT) is a growing tool in diagnosis and staging of prostate cancer. It is more sensitive for tumour detection than magnetic resonance imaging (MRI) reported according to the Prostate Imaging-Reporting and Data System (PI-RADS). Therefore, the combination of both imaging modalities may enhance diagnosis and tumour characterisation [1] to aid surgical planning and intra-operative orientation for prostate biopsy and radical prostatectomy. Potential clinical benefits are improved detection of clinically significant prostate cancer with prostate biopsy and reduced positive surgical margins during radical prostatectomy [2–4]. Although hybrid PET/MRI may provide combined imaging, PET/MRI machines are not widely available and have image resolution limitations. Conversely, image fusion of PSMA PET/CT and MRI may be a practical alternative that can be clinically integrated, however is labour intensive with questionable precision.

Registration algorithms are routinely used to perform image fusion of PSMA PET/CT and MRI in radiation oncology but not in urology, and thus clinical methods and values for alignment and registration errors rely on radiation therapy guidelines. Prostate clinical- and planning-target volumes (CTVs and PTVs) delineate the prostate and a 5–10 mm border around the prostate to account for uncertainties [5–7], respectively. It is important that uncertainties including patient positioning, acquisition times, time between imaging modalities and registration errors do not exceed the typical PTV boundary. In addition, it is well understood that compared with MRI, CT scans overestimate prostate CTVs [8]. As such, there has been extensive research investigating registration methods to accurately align CT and magnetic resonance (MR) images [8–20].

Several previous works have applied manual [8, 10, 11], semi-automated [9, 11–13, 16, 17, 19], automated rigid [14] and non-rigid [15, 18, 20] methods to perform MR-CT registration. Rigid prostate MR-CT registration studies have utilized point-based methods requiring manual placement of landmarks [8–13], iterative closest points between automatically identified landmarks [14], crude manual matching paired with automated intensity matching (using focused and non-focused regions of interest) [9], and automated voxel similarity methods measuring mutual information [11, 16]. In addition to rigid-only methods, Rivest-Henault and colleagues, developed a robust inverse-consistent algorithm combining both rigid and non-rigid techniques well suited to CT–MR alignment in prostate radiation therapy [15]. Similarly, Zhong et al. presented a combined rigid and deformable registration before their adaptable deformable registration method with finite element modelling [17]. Further deformable registration algorithms have utilized displacement vector fields [18], a probabilistic Bayesian framework [18], normalized mutual information [19] and a biomechanically constrained deep learning network [20]. However, previous works have not compared the accuracy and feasibility of clinical translation for a semi-automated clinical rigid registration technique with fast and easily explainable automated rigid and non-rigid registration techniques.

The hypothesis of the current study was that accuracy (according to volume and distance-based contour validation metrics) would be higher for the automated non-rigid registration method than automated rigid and semi-automated rigid methods. The purpose of the present study was to compare three types of registration processes: semi-automated clinical rigid registration, automated rigid, and non-rigid registration to quantify the accuracy associated within the CT-MR fusion process (initial step of PET/CT and MRI registration process) for urological care of prostate cancer and discuss the feasibility and accessibility of their integration into clinical practice.

## Methods

### Patient data

The present study analysed paired whole-pelvis MR and CT scan data from 20 prostate patients from Dowling *et al.’s* prior study [21]. The sequences used within this study were in line with PI-RADS standards. All patients were diagnosed with stage T1 to T3 tumours and intended to proceed with radiotherapy. Prior to the acquisition of MR and CT planning images, each patient had three pure gold fiducial markers inserted transrectally to assist with landmark localization [21]. These gold fiducial markers could be used for registration development. However, not all patients presenting for urological care of prostate cancer require or consent to the insertion of gold seeds. Within this paper, we wanted to present an accessible and easily explainable algorithm which can be broadly used across patients. Therefore, we chose to not use the gold seeds as part of the assessment in this paper.

The conventional planning CT scans (voxel size: 0.977 × 0.977 × 2.5 mm) were acquired with either a GE (Milwakee, USA) LightSpeed radiotherapy large bore scanner with 2.5 mm slices or a Toshiba (Tokyo, Japan) Acquilion scanner with 2.0 mm slices [21]. The MR images were acquired with a 2-dimensional axial T2-weighted turbo spin echo sequence (repetition time: 1400 ms, echo time: 97 ms, field of view: 200 mm, flip angle: 135°, voxel size: 0.625 × 0.625 × 2 mm) on a Siemens (Erlangen, Germany) Skyra 3T scanner [21]. For further information on the CT and MR protocol, bladder and bowel preparation, patient and MR coil positioning, refer to [21].

A manual contour of the prostate CTV was performed by an experienced radiation oncologist independently on the MR and CT image of each patient [21]. These manual contours were considered the gold standard and used in subsequent registration, prostate volume, and surface area analyses of the current study.

### Prostate contour registration

#### Semi-automated clinical practice registration

A clinician (urology registrar, 2 years clinical experience) used built-in automatic boxed-based registration tools to grossly and rigidly align the CT and MR images in two stages using commercial software (MIM Maestro *7.6.1*, MIM Software Inc, *USA*). Firstly, gross alignment was performed by selecting a region of interest (ROI) with bony landmarks, then a refined ROI was chosen around the soft-tissue of the prostate. Subsequently, manual translation adjustments were applied to improve the visual registration fitting. The output registration transform was used to project the moving CT prostate manual contour into the fixed MR image space using nearest neighbour interpolation with the 3D Slicer software (version 5.0.3) [22, 23].

### Automated rigid registration

Libraries within the Simple ITK package (version 2.1.1.2) were used to produce the automated rigid and non-rigid registration algorithms described below. Initially a negative normalized cross correlation image metric [24] was applied to the fixed MR- and moving CT-prostate manual contour. This correlation quantified the extent which the two images move in opposite directions. Subsequently, the gradient descent optimizer and rigid transformation [24] with additional scaling were applied to calculate the updated position of the moving contour iteratively. The alignment transformation was computed using linear interpolation [24] between the fixed MR- and moving CT-prostate manual contour. This transformation was used in combination with linear interpolation [24] to align the moving CT image with the fixed MR image. Subsequently, the calculated transform was applied with nearest neighbour interpolation [24] to register the moving CT prostate manual contour to the fixed MR manual contour. We note the automated rigid registration involved additional scaling to account for discrepancies in CT and MR prostate volumes reported previously in the literature. Herein, our automated rigid registration with scaling will be reported as the automated rigid registration.

### Automated non-rigid registration

The signed Maurer distance map filter [25] (implemented in [24]) was applied to the fixed MR manual contour and the rigidly registered CT manual contour. The fast symmetric forces demon’s registration filter [26] (implemented in [24]) was then computed on these distance map volumes to obtain a deformation field. The deformation field was applied with nearest neighbour interpolation to perform a grey-scale-based registration [24] between the fixed MR manual contour and the rigidly registered CT manual contour. Subsequently, the deformation field was used in combination with BSpline resampling to perform deformable registration [24] between the fixed MR image and the rigidly registered CT image.

### Prostate segmentation registration analyses

**Fig. 1:**
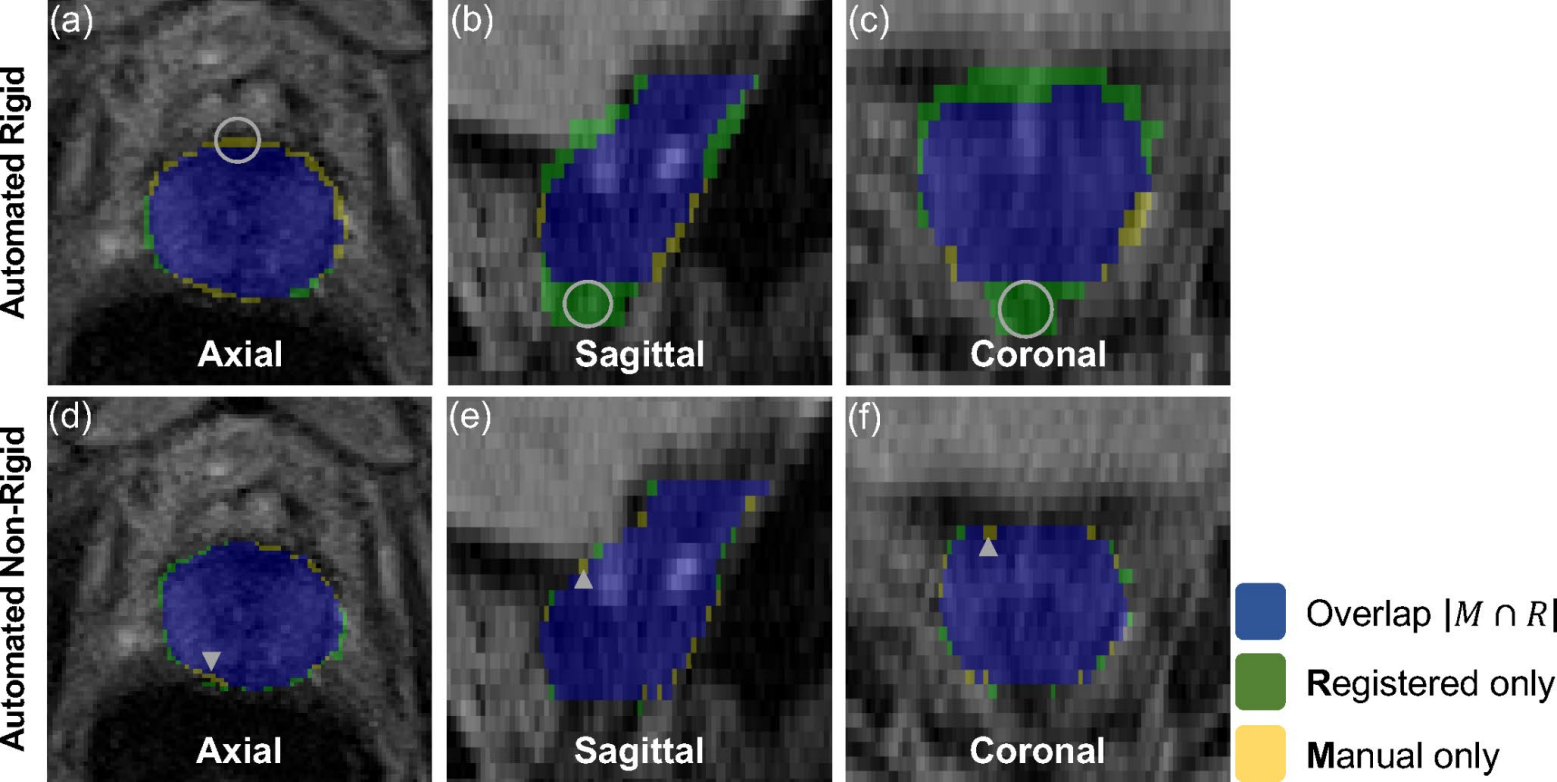
A representative view of the prostate contour overlap in each planar orientation (i.e., axial, sagittal and coronal) illustrates differences between the manual MR contours and the contours generated through the registration of the CT and MR images. The grey circles (row 1 (**a**-**c**)) and grey arrowheads (row 2 (**d**-**f**)) indicate the location of maximal HD (the largest minimum distance between the manual and registered contour) in each plane within the 3-dimensional image (i.e., axial (column 1), sagittal (column 2) and coronal (column 3)). The DSI measures the magnitude of overlap of two contours. M, manually contoured voxel; R; registered contour voxel; Blue label (M ∩ R), manual and registered contour overlap voxels; Green label (R), registered contour only voxels; Yellow label (M), manual contour only voxels; MR, magnetic resonance; CT, computed tomography; DSI, dice similarity index; HD, Hausdorff Distance

Manual contours of the prostate from MR examinations were used to assess each registration method. The aligned contours from each registration method were compared using the Dice similarity index (DSI) [27] for volume overlap (1 representing perfect overlap and 0 representing no overlap) and surface distance differences (mm) based on the 95% Hausdorff distance (HD) [28] and average surface distance (ASD) [29]. The HD is a measurement of the largest minimum distance between two contours. The 95% HD was used rather than the HD based on the sensitivity of the HD to outliers [28]. The ASD is the average distance calculated from the set of minimum distances between two contours. Figure 1 displays example illustrations of the DSI and HD measurements on an axial (column 1), sagittal (column 2) and coronal (column 3) plane within a 3-dimensional MR image of the prostate registered using an automated rigid (row 1) and non-rigid registration (row 2).

### Prostate segmentation overlap

Each registered binary label (generated from the CT manual contour) was threshold to a value not equal to 1. Subsequently, an image addition between the corresponding MR and threshold registered binary label produced an overlap of the two contours (where 1: MR only; 2: registered CT only; 3: MR and CT union). This overlap contour was used for subsequent qualitative assessment of each registration algorithm to accurately align the MR and CT contours. The assessment was performed on the prostate volume in three-dimensional space to visualize the number of mislabelled prostate voxels.

### Prostate surface generation and assessment

The manual (CT/ MR) and registered CT (semi-automated rigid/ automated rigid/ automated non-rigid) prostate manual contours were converted to surfaces using the marching cubes algorithm [30]. These surfaces were then smoothed using the windowed sinc algorithm [31] and used to calculate the prostate volume and surface area for comparison with previous reports of greater CT prostate volumes compared to those obtained using MR images [8]. In further analyses, the surface distance was calculated using the visualisation toolkit (VTK) [32] signed distance polydata filter between the registered CT contours and the MR contours to compare the alignment accuracy of different registration algorithms.

### Statistical analyses

Paired t-tests or Wilcoxon signed-rank tests [33] were used to observe comparisons for volume and surface area analyses of the CT and MR prostate surfaces. They were also applied to compare the performance of the semi-automated rigid, automated rigid and non-rigid registrations using surface- and distance-based metrics. Prior to performing the comparisons, the Shapiro-Wilk test was applied to check the normality of the data and Wilcoxon signed-rank tests were used if the data did not have a normal distribution. For the t-test analyses, Levene’s method was used to assess homogeneity of variances and Welch’s test was applied if samples did not have equal variances. Statistical significance was set *a priori* at *p* < 0.05 and all statistical analyses were calculated using a python package, SciPy (version 1.7.3) [34] .

## Results

On average, the semi-automated rigid-, automated rigid- and automated non-rigid-registration was completed within ~ 5 min, 1 min 38 s and 23 s, respectively.

### Registration comparison

**Fig. 2:**
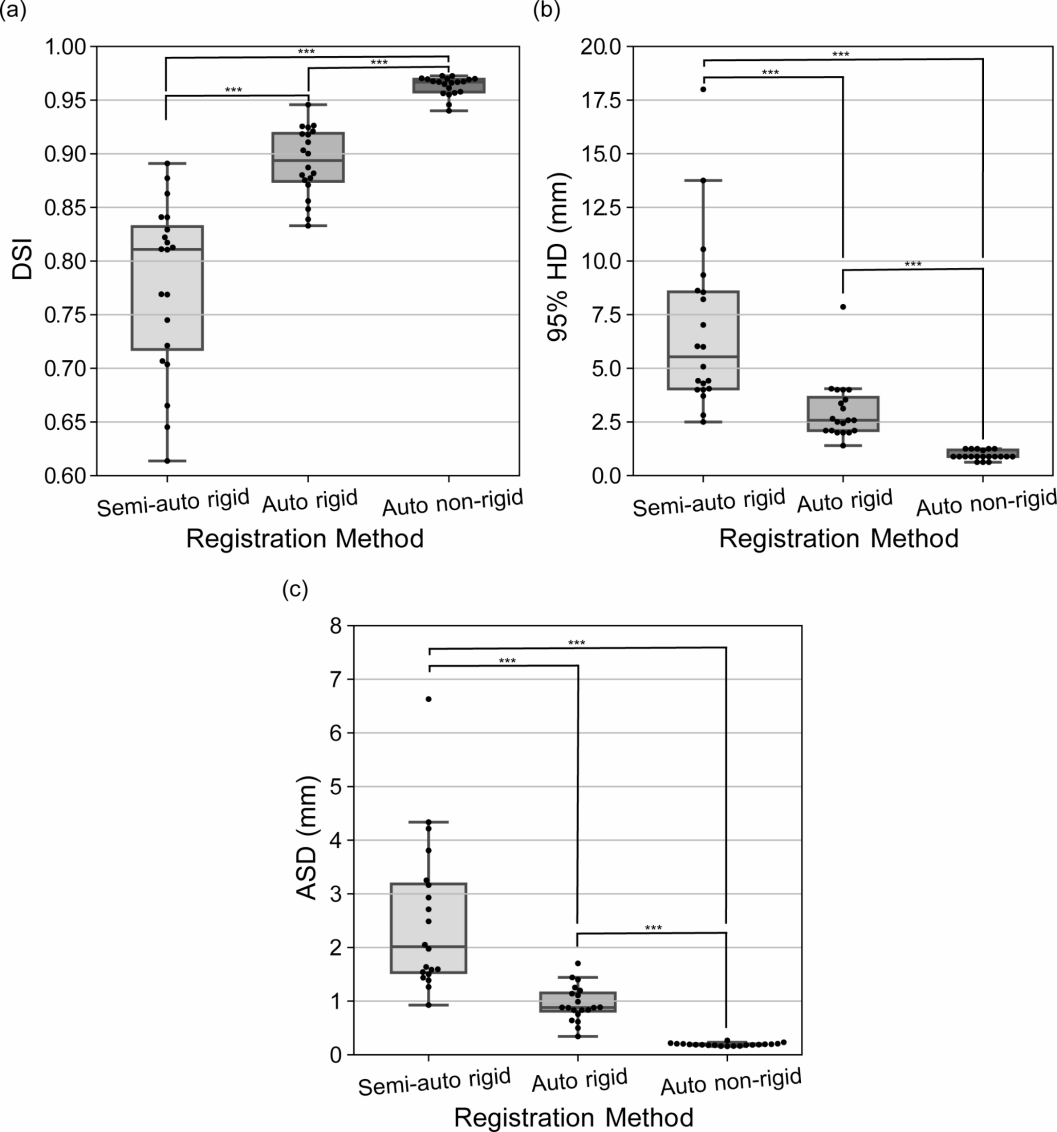
Boxplots of the evaluation metrics used in this study for comparisons between the semi-automated rigid-, automated rigid and non-rigid registration algorithm performance. (**a**) DSI values. (**b**) 95% HD values. (**c**) ASD values. The boxplot centreline marks the median value. DSI, dice similarity index; HD, Hausdorff distance; ASD, average surface distance; Auto, automated; ***, *p* < 0.001

Figure 2 displays boxplots, and Table 1 provides a summary of the mean and standard deviation data for the DSI (Fig. 2a), 95% HD (Fig. 2b) and ASD values (Fig. 2c) for the semi-automated rigid-, automated rigid- and automated non-rigid-registered prostate manual contours. Overall, the automated non-rigid approach had a significantly improved performance compared to the automated rigid- and semi-automated rigid-registration having better average scores and decreased spread for the DSI, 95% HD and ASD (all *p* < 0.001). Additionally, the automated rigid approach had a significantly improved performance compared to the semi-automated rigid-registration having better average scores and decreased spread for the DSI, 95% HD and ASD (all *p* < 0.001).

**Table 1:** Mean and standard deviation data for the semi-automated rigid-, automated rigid- and automated non-rigid-registration method in the DSI, 95% HD and ASD results

Registration Method	DSI	95% HD (mm)	ASD (mm)
Semi-automated rigid	0.778 ± 0.077	6.768 ± 3.189	2.522 ± 1.369
Automated rigid	0.892 ± 0.031	3. 018 ± 1.364	0.955 ± 0.325
Automated non-rigid	0.963 ± 0.009	0.951 ± 0.208	0.192 ± 0.025

DSI, dice similarity index; HD, Hausdorff distance; ASD, average surface distance

### Prostate segmentation overlap

**Fig. 3:**
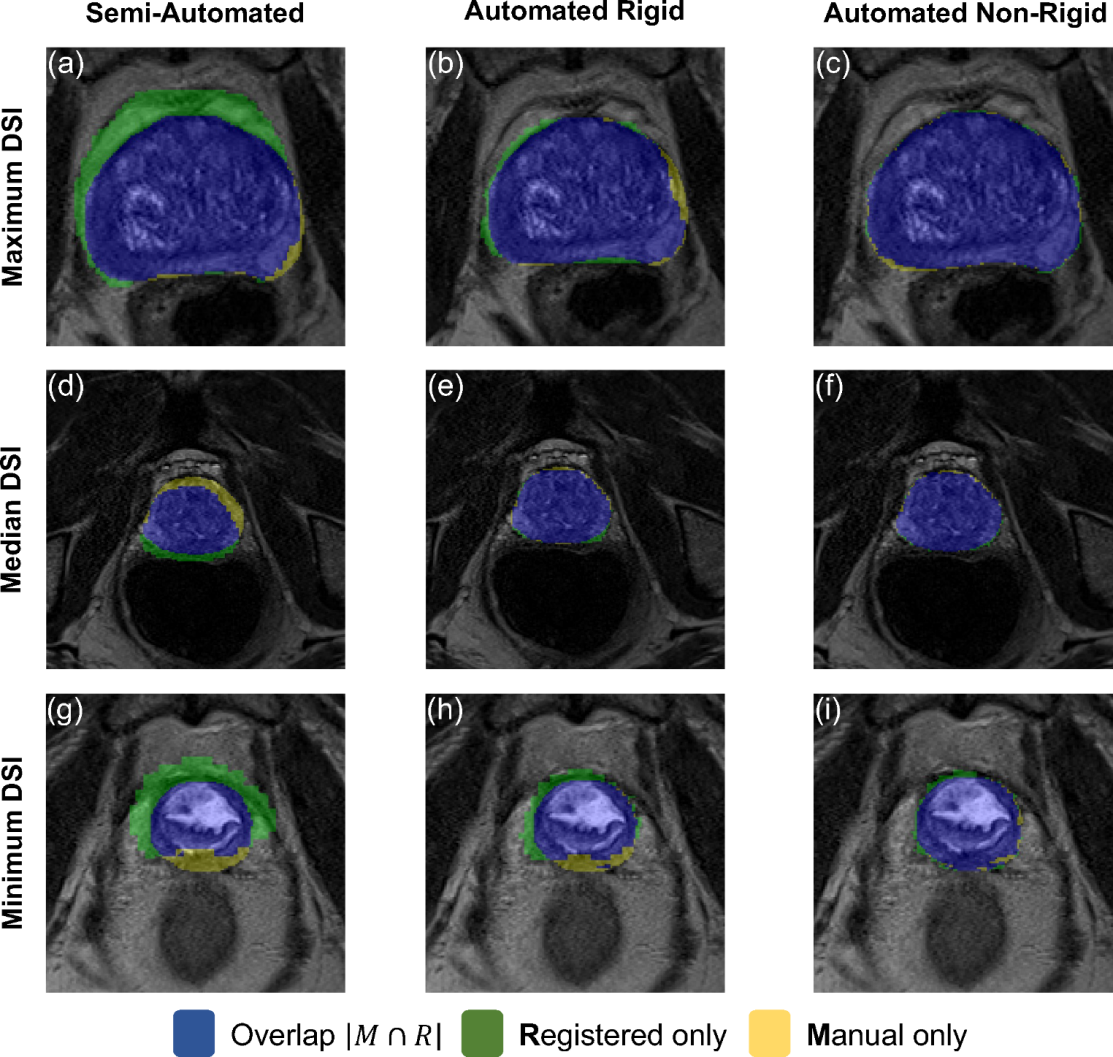
Axial view prostate contour overlap illustrations between the manual MR contours and the contours generated through the registration of the CT and MR images. Columns 1 (**a, d, g**), 2 (**b, e, h**) and 3 (**c, f, i**) display the semi-automated rigid-, automated rigid- and automated non-rigid-registered results, respectively. Rows 1 (**a-c**) , 2 (**d-f**) and 3 (**g-i**) display the case and prostate region which achieved the maximum-, median- and minimum-DSI result using the automated non-rigid registration method, respectively. Blue label, manual and registered contour overlap voxels; Green label, registered contour only voxels; Yellow label, manual contour only voxels; MR, magnetic resonance; CT, computed tomography; DSI, dice similarity index

Figure 3 displays axial view illustrations of the overlap between the manual prostate contour completed on the MR examination and the CT contour registered using the semi-automated rigid-, automated rigid- and automated non-rigid-registration method. This visualization clearly shows the significantly improved fitting (shaded in blue) of the CT contour using the automated rigid (column 2) and automated non-rigid registration (column 3) compared to the semi-automated rigid registration method (column 1). The cases displayed obtained the highest (row 1), median (row 2) and lowest DSI (row 3) reported using the automated non-rigid registration method. As such, the cases in this figure are representative of the contour results within the mid prostate region obtained from the full cohort. Visual assessments across axial prostate slices revealed the overlap of manual and registered contour results appeared to be highest in the mid prostate and lower in the ends of the prostate gland (apex and base). Within all cases, the CT contour registered using an automated non-rigid algorithm had an excellent fitting to the manual contour.

### Prostate volume delineated from MR and CT examinations

**Fig. 4:**
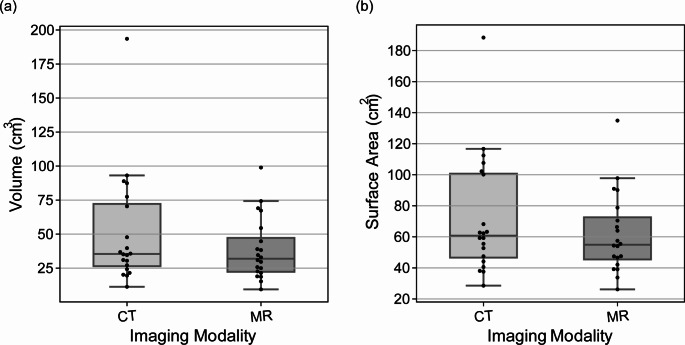
Original CT and MR image manually contoured prostate volumes (**a**) and surface areas (**b**). CT, computed tomography; MR, magnetic resonance

Figure 4 shows boxplots for the manually contoured CT and MR volume (Fig. 4a) and surface area (Fig. 4b). Compared to the MR examinations, the CT examinations obtained a significantly larger median prostate volume (35.40 cm^3^ v 31.89 cm^3^, *p* = 0.005) and a non-significantly larger median surface area (60.77 cm^2^ v 54.94 cm^2^, *p* = 0.052). On average the manual prostate segmentations had a CT/ MR volume of 51.28 ± 40.95/ 38.54 ± 22.61 cm^3^. On average the manual prostate segmentations had a CT/ MR surface area of 72.36 ± 37.40/ 61.83 ± 25.47 cm^2^.

### Prostate surface assessment

**Fig. 5:**
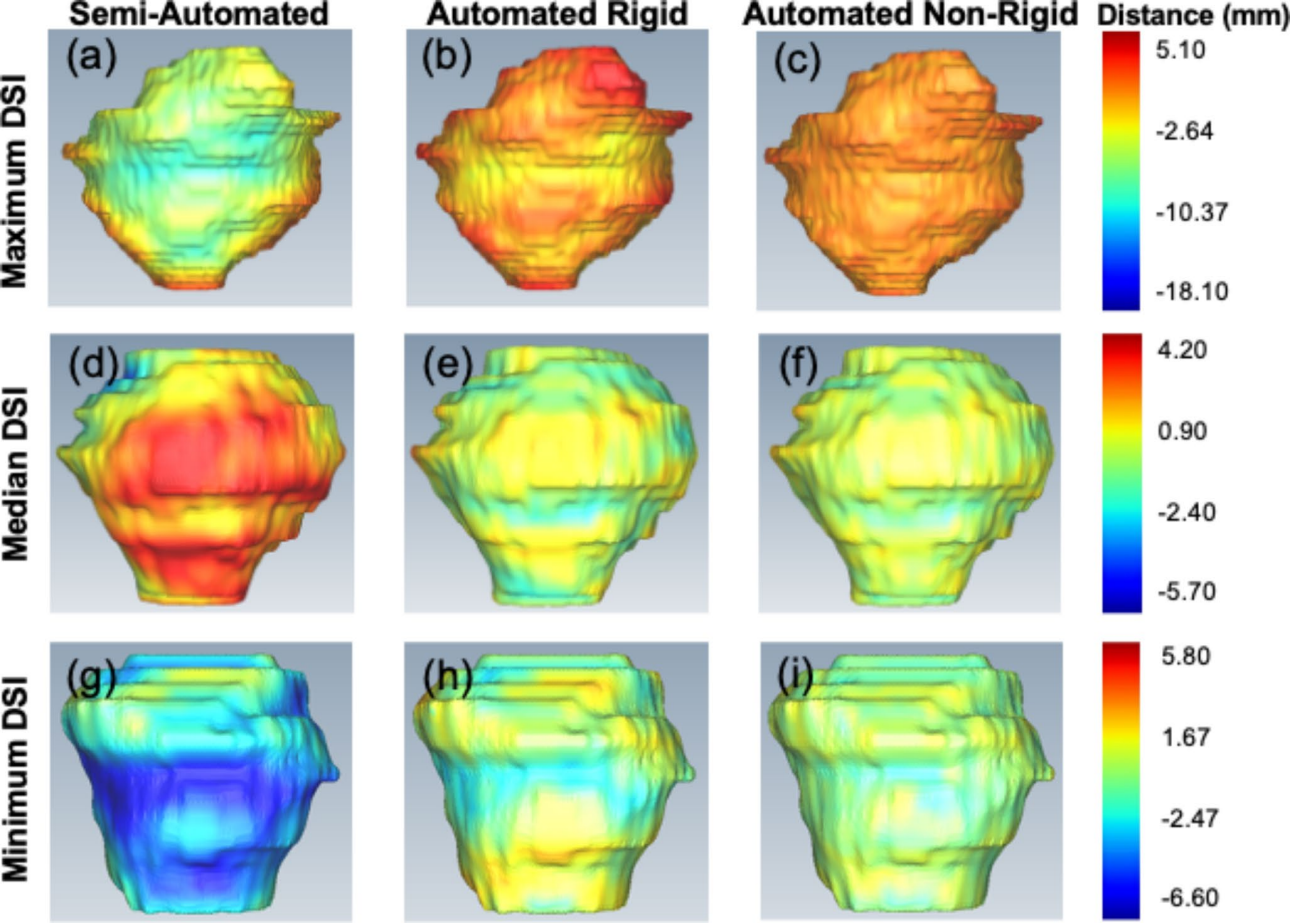
Coronal view prostate surface distance between the manual MR contours and the contours generated through the registration of the CT and MR images. Columns 1 (**a, d, g**), 2 (**b, e, h**) and 3 (**c, f, i**) display the semi-automated rigid-, automated rigid- and automated non-rigid-registered results, respectively. Rows 1 (**a-c**), 2 (**d-f**) and 3 (**g-i**) display the case which achieved the maximum-, median- and minimum DSI result using the automated non-rigid registration method, respectively. The signed distance scalar bar (rainbow colour map on right side of each row) has units in mm. MR, magnetic resonance; CT, computed tomography; DSI, dice similarity index

Each prostate surface generated from the semi-automated rigid-, automated rigid- and non-rigidly-registered contour was compared with the manual MR generated surface separately using the VTK signed distance. Figure 5 displays selected coronal view CT prostate surfaces generated after semi-automated rigid- (column 1), automated rigid- (column 2) and automated non-rigid-registration (column 3) with the MR image. This visualization clearly shows the significantly improved surface-based fitting of the CT contour using the automated rigid and automated non-rigid registration compared to the semi-automated rigid registration method. The cases displayed obtained the highest (row 1), median (row 2) and lowest DSI (row 3) reported using the automated non-rigid registration method. Within all cases assessed, the CT contour registered using an automated non-rigid algorithm had an excellent fitting to the manual MR contour.

## Discussion

The current study described and assessed three different CT-MR registration techniques which utilized semi-automated rigid-, automated rigid- and automated non-rigid-techniques. Overall, feasibility of all three methods is shown by sufficient registration accuracy on image stacks of typical clinical quality that occurred within acceptable timeframes (23 s to ~ 5 min). The semi-automated rigid-registration method is accessible now via standard software while the higher accuracy automated non-rigid method uses an accessible and easily explainable algorithm that could benefit from pairing with an automated segmentation technique.

When comparing the three registration techniques, the automated non-rigid approach had a significantly improved performance compared to the automated rigid- and semi-automated rigid-registration. While the automated rigid approach had a significantly improved performance compared to the semi-automated rigid-registration. Manual and registered contour overlap- and prostate surface distance-visualizations revealed a trend for increasing accuracy from the semi-automated rigid-, automated rigid- to the automated non-rigid registration results. As expected, compared to the MR examinations, the CT examinations obtained a significantly larger median prostate volume and a non-significantly larger median surface area. On average, the semi-automated rigid-, automated rigid- and automated non-rigid-registration was completed within ~ 5 min, 1 min 38 s and 23 s, respectively.

In the present study, quantitative and qualitative analyses of three different CT-MR prostate registration algorithms, revealed that automated non-rigid registration outperforms both automated rigid- and semi-automated rigid-registration methods. The automated non-rigid registration achieved excellent average DSI (0.963 ± 0.009), 95% HD (0.951 ± 0.208) and ASD (0.192 ± 0.025) values (Fig. 2). The high degree of agreement between the manual and automatically registered prostate contours in the present work compared favourably with previous prostate CT-MR registration studies using manual [8, 12], semi-automated [16] and automated [14, 15, 18–20, 35] methods (Table S1). Individual qualitative assessments of the automated non-rigidly registered contour intersection with the original manual contour showed almost perfect overlap within patients that were reported to have the minimum, median and maximum DSI value of the full cohort (Fig. 3). Furthermore, the highest overlap of the registered and manual contour was present at the mid prostate and lowest at the prostate ends (apex and base) which is consistent with studies evaluating segmentation of zonal anatomy of prostate. This is likely due to the prostatic apex having a similar intensity profile to surrounding structures on a T2-weighted sequence, and the prostatic base transitioning from the bladder neck [36]. Additional qualitative comparisons between the manual and registered prostate volume showed distinct improvements in the signed distance values with the application of the automated registration algorithms in comparison to that requiring manual intervention (Fig. 5). This visual inspection supported the application of the automated non-rigid registration algorithm reported within this study to achieve a globally accurate prostate surface fitting.

The relatively poorer DSI, 95% HD and ASD achieved with the semi-automated rigid registration could be at least partially attributed to the significant (*p* = 0.005) prostate volumetric difference between the CT (51.28 ± 40.95 cm^3^) and MR (38.54 ± 22.61 cm^3^) acquired scans (Fig. 4, Table S1). In agreement with these results, previous works have reported the prostate CTV delineated on CT images can be up to 171% of the prostate CTV delineated on an MR image [5, 8, 37]. In addition, earlier MR and CT studies assessing prostate volume are in good general agreement with our study where mean volumes delineated on MR and CT images of 30.8 to 33.0 cm^3^ and 46.0 to 46.5 cm^3^ have been calculated, respectively [8, 10]. The automated non-rigid and rigid registration within the current study applied a scaling step to account for the known prostate volume discrepancy between CT and MR acquired scans of the same patient. Without this scaling step, scan-specific volume differences can create registration inaccuracies (e.g., those reported from our semi-automated rigid registration). A comparison between the semi-automated and automated rigid (plus scaling) method within the current paper can be used as an indication of the effect of scaling. The substantial differences between the current study CTV’s and the GTV’s in Ilamurugu et al.’s [9] (CT: 22.11 cm^3^; MR: 17.52 cm^3^) may be due to considerable differences in the size of the prostates in these cohorts.

Differences in internal pelvic anatomy such as the bladder and rectum have been identified to create a prostate bed tilt [38] and variability in the prostate position [39, 40]. Bladder and rectum filling can alter the prostate target volume position, which leads to difficult image registration. In radiation therapy, there are full bladder and empty rectum protocols to better control prostate position and reduce radiation exposure risk outside the PTV, however even these protocols increase bladder size variability [41, 42] and an empty rectum is hard to replicate. Furthermore, despite strict adherence to this imaging protocol, substantial bladder volume changes can occur. In addition to these anatomical variations, CT and MR images observe significant differences in the prostate size [8, 10, 19]. Although image registration may have a different type of role in urology through pre-operative planning and intra-operative guidance, non-rigid registration can be utilized to perform a deformation-based alignment of the images of interest to accommodate for these anatomical differences and imaging modality variabilities.

The current study presented a robust, fast, and easily explainable automated non-rigid registration algorithm ideal for usage in a clinical setting. This non-rigid algorithm used an anatomical constraint (i.e., the MR contour) to preserve the real prostate morphology during deformations. The limits of stretching for the CT morphology are bound by the MR contour and the intensity of deformations depends on the similarity between the MR and CT contour. In addition, although the CT contour can be overestimated, our application of the MR contour as fixed reduced the potential for discrepancies in the preservation of real morphology. The planning CT scan used in the current study had an equivalent quality (i.e., not a high dose and no contrast applied) to the CT component of clinical PET/CT acquisitions. As such, the verification of the automated non-rigid registration algorithm within this study is transferrable to potential PET/CT clinical applications. It is also important for automated registration algorithms to be explainable to ensure their future integration within the clinic. If a clinician can understand an algorithm, they are more likely to trust the results it provides and adopt it within their workflow. However, prior to the adoption of the presented algorithm within the clinical workflow, a validation study should be conducted in a prospective clinical trial. In addition, future work could be conducted to develop an automated end-to-end prostate (e.g., lesion, CTV, PTV) segmentation and registration pipeline for paired CT-MR images. Previous automated MR prostate segmentation studies have reported very promising results [43–48]. The combination of a previously developed automated segmentation algorithm with the presented registration algorithm may reduce costs and image post-processing time, increase prostate contour and image alignment accuracy, to potentially improve prostate cancer diagnosis and management.

Although automated non-rigid registration is the most accurate method, all three registration methods demonstrate an accuracy level which is consistent with literature and complies with the recommendations in general [49]. Semi-automated rigid registration, performed on clinical software available in hospitals is thus a quick (performed in ~ 5 min), feasible, and accessible option currently to deliver CT-MRI registration. This image registration can lead to PSMA PET/CT and MRI fusion, which can be utilised for prostate cancer care. The automated registration methods are however promising given their superior accuracy measures and lack of inter user variability. Once automated prostate volume segmentation is integrated into the automated registration algorithm, we believe they may be ready for verification in a clinical setting and for future clinical application.

There are several limitations to this study. First, a relatively small sample of 20 patients were assessed. Future works are required to confirm the conclusions made in the current study. Second, the patients within this study did not have any PSMA PET images acquired. Therefore, additional qualitative analyses assessing the utility of the registration algorithms presented in this study for the diagnosis and staging of prostate cancer using PSMA could not be conducted. Third, the MR images used in the present study were acquired using a T2 sequence on a Siemens (Erlingen, Germany) Skyra 3T scanner [21]. Future research should explore the utility of the automated registration algorithms presented when applied to other 3D MR sequences such as diffusion-weighted-, dynamic contrast-enhanced- [50], and T1-weighted-imaging which have been reported to have a high diagnostic accuracy for prostate cancer [51]. Finally, the patients within this study did not undergo radical prostatectomy to remove any cancerous tissues. As such, there is no ground truth histopathology for verification of accuracy. In addition, the combination of PSMA and MRI imaging to aid in surgical planning, intra-operative orientation, and potentially improve clinical outcomes through reducing positive margins could not be assessed, which also leads to the inability of quantifying the margin of error of image registration in urological practice. It is expected that future investigations will evaluate the utility of the automated non-rigid registration algorithm to aid in pre-operative planning, intra-operative positioning, and post-operative patient outcomes.

## Conclusion

All three CT/MRI registration techniques studied here demonstrated sufficient accuracy for exploring their clinical use. The fully automated non-rigid registration algorithm presented in the current study outperformed the semi-automated rigid registration method commonly used in clinical practice and an automated rigid registration technique. Currently, the semi-automated rigid registration is a quick, feasible, and accessible method to perform image registration for prostate cancer care by urologists and radiation oncologists.

### Electronic supplementary material

Below is the link to the electronic supplementary material.

Supplementary Material 1: **Table S1** Comparison of the present study with related works on prostate CT-MR registration

## List of abbreviations

PSMA: Prostate specific membrane antigen
PET: Positron emission tomography
CT: Computed tomography
MRI: Magnetic resonance imaging
CTV: Clinical target volume
PTV: Planning target volume
ROI: Region of interest
DSI: Dice similarity index
HD: Hausdorff distance
ASD: Average surface distance
VTK: Visualisation toolkit
