# Supplementary material for: Prostate volume analysis in image registration for prostate cancer care: a verification study
Source: Phys Eng Sci Med. 2023 Oct 11;46(4):1791–802. doi: 10.1007/s13246-023-01342-4 (PMC10703743; doi:10.1007/s13246-023-01342-4)
Supplement: Supplementary file 1 — Supplementary Material 1: Table S1 Comparison of the present study with related works on prostate CT-MR registration [file 13246_2023_1342_MOESM1_ESM.docx]

# **Supplementary Material**

## **Table S1** Comparison of the present study with related works on prostate CT-MR registration.

| Study | Registration Description | N | MRI Strength (T) | Accuracy Measure  Average ± SD | Average CTV_CT_ (cm^3^) | Average CTV_MR_ (cm^3^) |
| --- | --- | --- | --- | --- | --- | --- |
| [8] | Manual rigid: fiducial markers | 10 | 1.5 | DSI 0.79 ± 0.6 | 46.5 ± 13.9 | 33.0 ± 11.2 |
| [10] | Manual rigid: fiducial markers | 15 | 3 | - | 46.0 | 30.8 |
| [13] | Semi-auto rigid: fiducial markers | 29 | ND | Dose distribution: CTV_30 (seminal vesicle) D99% for all N, CTV_35 (prostate corpus) for 97% N, GTV_50 (local boost) for 83% N |  |  |
| [12] | Semi-auto rigid: point match | 42 | 3 | DSI 0.94 (median) | - | - |
| [9] | Semi-auto rigid: point match | 30 | 1.5 | - | GTV: 22.11 | GTV: 17.52 |
| [11] | manual rigid: fiducial markers compared to semi-auto rigid: MI | 30 | 1.5 | DSI 0.87 ± 0.05 | - | - |
| [17] | Semi-auto non-rigid: finite element model, B-spline | 10 | 1 | Average prostate centroid deviation: 0.28 cm | - | - |
| [16] | Semi-auto non-rigid: B-spline, MI | 5 | ND | DSI: 0.75-0.88 (CT-T1w); 0.72-0.76 (CT-T2w) | - | - |
| [14] | Auto rigid: seed segmentation; iterative closest points | 24 | 3 | Average localization error: 0.8 ± 0.8 mm | - | - |
| [15] | Auto: rigid/ non-rigid: block matching, inverse-consistent point set alignment. | 35 | 3 | DSI 0.64 ± 0.12 | - | - |
| [18] | Auto non-rigid: normalized MI with inverse-consistent  diffeomorphic | 10 | 3 | Translational vector differences: 0.37 ± 0.23 mm; rotational differences: < 1.1° in all 3 directions | - | - |
| [35] | Auto non-rigid: normalized MI and seed matching | 12 | ND | MI-seed match translation differences (mm) of 0.6 right–left, -0.5 posterior-anterior, -1.2 inferior–superior | - | - |
| [19] | Auto non-rigid: normalized MI | 12 | 1.5 | DSI 0.84 (median) | 45.5 (median) | 38.1 (median) |
| [20] | Auto non-rigid: point cloud matching network | 50 | 1.5 | DSI 0.93 ± 0.01 | - | - |
| Present Study | Semi-auto rigid  Auto rigid: negative normalized cross correlation  Auto non-rigid: fast symmetric forces demon’s | 20 | 3 | DSI 0.778 ± 0.077  DSI 0.892 ± 0.031  DSI 0.963 ± 0.009 | Manual: 51.28 ± 40.95 | Manual: 38.54 ± 22.61 |

N, subjects; MRI, magnetic resonance imaging; T, tesla; DSI, dice similarity index; SD, standard deviation; CTV_CT_, clinical target volume on computed tomography; CTV_MR_, clinical target volume on magnetic resonance image; GTV, gross tumour volume; Auto, automated; ND, not disclosed; MI, mutual information.
